# 1-(2,5-Dimethyl­phen­yl)piperazine-1,4-diium tetra­chloridozincate monohydrate

**DOI:** 10.1107/S1600536811007562

**Published:** 2011-03-09

**Authors:** Riadh Kefi, Frédéric Lefebvre, Matthias Zeller, Cherif Ben Nasr

**Affiliations:** a Laboratoire de Chimie des Matériaux, Faculté des Sciences de Bizerte, 7021 Zarzouna, Tunisia; b Laboratoire C2P2 (COMS group), École Supérieure de Chimie Physique Électronique, Villeurbanne, France; c Youngstown State University, Department of Chemistry, One University Plaza, Youngstown, Ohio 44555-3663, USA

## Abstract

In the title compound, (C_12_H_20_N_2_)[ZnCl_4_]·H_2_O, the two piperazine N atoms are protonated and the [ZnCl_4_]^2−^ anions adopt a slightly distorted tetra­hedral configuration. In the crystal, O—H⋯Cl hydrogen bonds link the tetra­chloridozincate anions and the water mol­ecules into corrugated inorganic chains parallel to [010]. The crystal structure is stabilized by N—H⋯Cl, N—H⋯O and O—H⋯Cl hydrogen bonds, with the N—H hydrogen bond originating from one of the two N atoms being trifurcated.

## Related literature

For common applications of organic–inorganic hybrid materials, see: Dai *et al.* (2002 ▶); Tao *et al.* (2003 ▶). For a related structure and discussion of geometrical features, see: Ben Gharbia *et al.* (2007 ▶). For the geometry around the zinc atom, see: Harrison (2005 ▶).
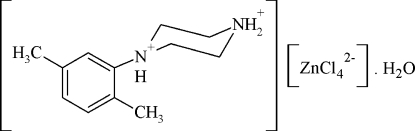


## Experimental

### 

#### Crystal data

(C_12_H_20_N_2_)[ZnCl_4_]·H_2_O
*M* *_r_* = 417.49
Monoclinic, 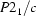

*a* = 7.0999 (8) Å
*b* = 8.0679 (8) Å
*c* = 29.933 (3) Å
β = 95.314 (2)°
*V* = 1707.2 (3) Å^3^
*Z* = 4
Mo *K*α radiation
μ = 2.06 mm^−1^
*T* = 100 K
0.45 × 0.39 × 0.31 mm

#### Data collection

Bruker SMART APEX CCD diffractometer
Absorption correction: multi-scan (*SADABS*; Bruker, 2009 ▶) *T*_min_ = 0.589, *T*_max_ = 0.746
10440 measured reflections
4995 independent reflections
4738 reflections with *I* > 2σ(*I*)
*R*_int_ = 0.016

#### Refinement

*R*[*F* ^2^ > 2σ(*F* ^2^)] = 0.027
*wR*(*F* ^2^) = 0.064
*S* = 1.13
4995 reflections
194 parameters
3 restraints
H atoms treated by a mixture of independent and constrained refinement
Δρ_max_ = 0.56 e Å^−3^
Δρ_min_ = −0.44 e Å^−3^

### 

Data collection: *APEX2* (Bruker, 2009 ▶); cell refinement: *SAINT* (Bruker, 2009 ▶); data reduction: *SAINT*; program(s) used to solve structure: *SHELXTL* (Sheldrick, 2008 ▶); program(s) used to refine structure: *SHELXTL*; molecular graphics: *SHELXTL*; software used to prepare material for publication: *SHELXTL*.

## Supplementary Material

Crystal structure: contains datablocks global, I. DOI: 10.1107/S1600536811007562/vn2004sup1.cif

Structure factors: contains datablocks I. DOI: 10.1107/S1600536811007562/vn2004Isup2.hkl

Additional supplementary materials:  crystallographic information; 3D view; checkCIF report

## Figures and Tables

**Table 1:** Hydrogen-bond geometry (Å, °)

*D*—H⋯*A*	*D*—H	H⋯*A*	*D*⋯*A*	*D*—H⋯*A*
N1—H1*A*⋯Cl3^i^	0.90 (2)	2.46 (2)	3.2300 (14)	144 (2)
N2—H2*A*⋯O1	0.92	1.92	2.7925 (17)	157
N2—H2*B*⋯O1^ii^	0.92	2.19	2.9145 (17)	135
N2—H2*B*⋯Cl4^ii^	0.92	2.77	3.3965 (14)	127
N2—H2*B*⋯Cl1^iii^	0.92	2.85	3.4036 (14)	120
O1—H1*C*⋯Cl2	0.83 (2)	2.43 (2)	3.2361 (12)	164 (2)
O1—H1*B*⋯Cl4^iii^	0.84 (2)	2.31 (2)	3.1518 (13)	178 (3)

Symmetry codes: (i) 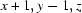; (ii) 
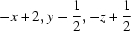
; (iii) 
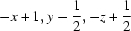
.

**Fig. 1.**
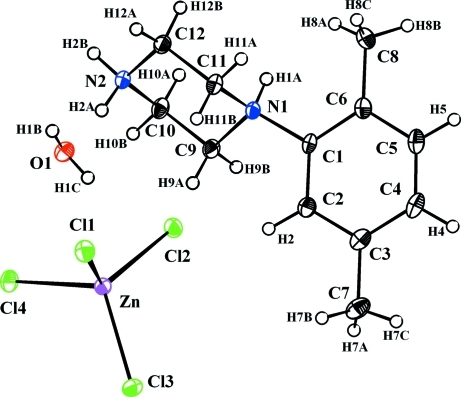
A view of the title compound, showing 50% probability displacement ellipsoids, arbitrary spheres for the H atoms, and the atom numbering scheme.

**Fig. 2.**
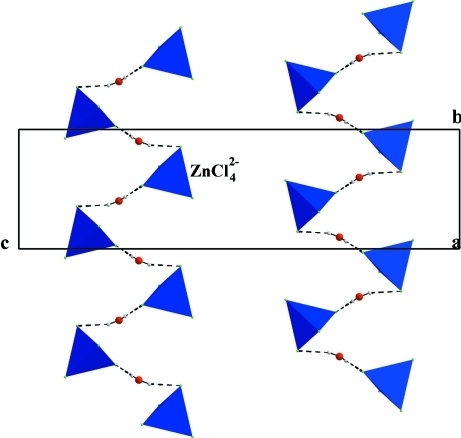
Projection along the a axis of the inorganic corrugated chain in the structure of the title compound. Hydrogen bonds are denoted as dashed lines.

**Fig. 3.**
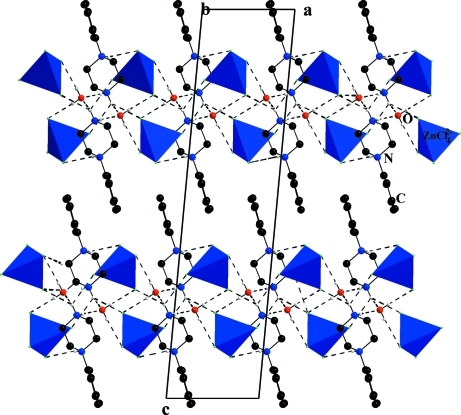
The packing of [1-(2,5-(CH3)2C6H3)C4H11N2]ZnCl4.H2O, viewed down the b axis. Hydrogen bonds are denoted by dashed lines.

## supplementary crystallographic information

### Comment

Organic-inorganic hybrid materials continue to attract much attention due to their potential applications in various fields (Dai *et al.*, 2002; Tao *et al.*, 2003). In this work, we report the crystal structure of one such compound, [1-(2,5-(CH_3_)_2_C_6_H_3_)C_4_H_11_N_2_]ZnCl_4_.H_2_O. As shown in Fig. 1, the asymmetric unit consists of one 1-(2,5-dimethyphenyl)piperazine-1,4-diium dication doubly protonated at the N1 and N2 nitrogen atoms, one water molecule and one [ZnCl_4_]^2-^ anion. The atomic arrangement of [1-(2,5-(CH_3_)_2_C_6_H_3_)C_4_H_11_N_2_]ZnCl_4_.H_2_O can be described as built up by corrugated inorganic chains of [ZnCl_4_]^2-^ tetrahedra and water molecules that extend along the *b* axis, held together by O—H···Cl hydrogen bonds (Fig. 2, Table 1). Two such chains cross the unit cell at *z* = (2*n* +1)/4 and *x* = 1/2. The organic groups are located between these chains and connect to them through N—H···Cl and N—H···O hydrogen bonds to form a three dimensional infinite network (Fig. 3, Table 1). Among all the hydrogen bonds, one is trifurcated: N2—H2B···(Cl1, Cl4, O1). Within the network, the 1-(2,5-dimethylphenyl)piperazine-1,4-diium dications are arranged into antiparallel dimers. No π-π stacking interactions between the phenylene rings or C—H···π interactions towards them are observed. In the organic entity, the piperazine-1,4-diium ring adopts a typical chair conformation and all the geometrical features agree with those found in 1-(2,3-dimethylphenyl)piperazinium tetrachlorozincate(II) monohydrate (Ben Gharbia *et al.*, 2007). It is worth noticing that in the [ZnCl_4_]^2-^ anion, the Zn—Cl bond lengths and Cl—Zn—Cl bond angles are not equal to one another, but vary with the environment around the Cl atom with Zn—Cl bond lengths between 2.2619 (4) and 2.2857 (4) Å. In the title compound, all the chloride ions are involved in hydrogen bonding. However, only the Cl4 chloride atom participates in two N—H···Cl bonds, and the Zn1—Cl4 bond distance is with 2.2857 (4) Å the longest (Table 1). The Cl—Zn—Cl angles range between 105.803 (16) and 112.737 (17)°. These values indicate that the coordination geometry of the Zn atom can be regarded as being a slightly distorted tetrahedron (Harrison, 2005).

### Experimental

A mixture of an aqueous solution of 1-(2,5-dimethyphenyl)piperazine (2 mmol, 0.380 g), zinc chloride (2 mmol, 0.396 g) and HCl (10 ml, 0.4 *M*) in a Petri dish was slowly evaporated at room temperature. Single crystals of the title compound, suitable for X-ray diffraction analysis, were obtained after several days by slow evaporation at room temperature (yield 68%).

### Refinement

Reflection (0 0 1) was obscured by the beamstop and was omitted from the refinement. C—H hydrogen atoms were placed in calculated positions with C—H distances in the range 0.93–0.97 Å. The water hydrogen atom positions were refined with O—H distance restraints of 0.84 (2) Å, and the N—H distance of N1 to 0.91 (2) Å. The *U*_iso_(H) values of all H atoms were constrained to 1.2 or 1.5 times *U*_eq_ of the respective parent atom.

### Crystal data

**Table:**

(C_12_H_20_N_2_)[ZnCl_4_]·H_2_O	*F*(000) = 856
*M**_r_* = 417.49	*D*_x_ = 1.624 Mg m^−^^3^
Monoclinic, *P*2_1_/*c*	Mo *K*α radiation, λ = 0.71073 Å
Hall symbol: -P 2ybc	Cell parameters from 6435 reflections
*a* = 7.0999 (8) Å	θ = 2.6–31.0°
*b* = 8.0679 (8) Å	µ = 2.06 mm^−^^1^
*c* = 29.933 (3) Å	*T* = 100 K
β = 95.314 (2)°	Block, colourless
*V* = 1707.2 (3) Å^3^	0.45 × 0.39 × 0.31 mm
*Z* = 4	

### Data collection

**Table:**

Bruker SMART APEX CCD diffractometer	4995 independent reflections
Radiation source: fine-focus sealed tube	4738 reflections with *I* > 2σ(*I*)
graphite	*R*_int_ = 0.016
ω scans	θ_max_ = 31.0°, θ_min_ = 2.6°
Absorption correction: multi-scan (*SADABS*; Bruker, 2009)	*h* = −7→10
*T*_min_ = 0.589, *T*_max_ = 0.746	*k* = −11→9
10440 measured reflections	*l* = −43→35

### Refinement

**Table:**

Refinement on *F*^2^	Primary atom site location: structure-invariant direct methods
Least-squares matrix: full	Secondary atom site location: difference Fourier map
*R*[*F*^2^ > 2σ(*F*^2^)] = 0.027	Hydrogen site location: inferred from neighbouring sites
*wR*(*F*^2^) = 0.064	H atoms treated by a mixture of independent and constrained refinement
*S* = 1.13	*w* = 1/[σ^2^(*F*_o_^2^) + (0.0258*P*)^2^ + 1.2487*P*] where *P* = (*F*_o_^2^ + 2*F*_c_^2^)/3
4995 reflections	(Δ/σ)_max_ = 0.001
194 parameters	Δρ_max_ = 0.56 e Å^−^^3^
3 restraints	Δρ_min_ = −0.44 e Å^−^^3^

### Special details

**Table:**

Geometry. All e.s.d.'s (except the e.s.d. in the dihedral angle between two l.s. planes) are estimated using the full covariance matrix. The cell e.s.d.'s are taken into account individually in the estimation of e.s.d.'s in distances, angles and torsion angles; correlations between e.s.d.'s in cell parameters are only used when they are defined by crystal symmetry. An approximate (isotropic) treatment of cell e.s.d.'s is used for estimating e.s.d.'s involving l.s. planes.
Refinement. Refinement of *F*^2^ against ALL reflections. The weighted *R*-factor *wR* and goodness of fit *S* are based on *F*^2^, conventional *R*-factors *R* are based on *F*, with *F* set to zero for negative *F*^2^. The threshold expression of *F*^2^ > σ(*F*^2^) is used only for calculating *R*-factors(gt) *etc*. and is not relevant to the choice of reflections for refinement. *R*-factors based on *F*^2^ are statistically about twice as large as those based on *F*, and *R*- factors based on ALL data will be even larger.

### Fractional atomic coordinates and isotropic or equivalent isotropic displacement parameters (Å^2^)

**Table:**

	*x*	*y*	*z*	*U*_iso_*/*U*_eq_	
C1	0.8731 (2)	0.27945 (19)	0.07313 (5)	0.0113 (3)	
C2	0.8347 (2)	0.4334 (2)	0.05374 (5)	0.0130 (3)	
H2	0.8573	0.5314	0.0710	0.016*	
C3	0.7627 (2)	0.4442 (2)	0.00884 (5)	0.0141 (3)	
C4	0.7332 (2)	0.2967 (2)	−0.01512 (5)	0.0165 (3)	
H4	0.6897	0.3009	−0.0461	0.020*	
C5	0.7664 (2)	0.1440 (2)	0.00543 (5)	0.0167 (3)	
H5	0.7415	0.0460	−0.0117	0.020*	
C6	0.8351 (2)	0.1302 (2)	0.05048 (5)	0.0132 (3)	
C7	0.7193 (2)	0.6104 (2)	−0.01244 (6)	0.0181 (3)	
H7A	0.8379	0.6673	−0.0171	0.027*	
H7B	0.6471	0.6773	0.0073	0.027*	
H7C	0.6448	0.5950	−0.0414	0.027*	
C8	0.8579 (2)	−0.0383 (2)	0.07188 (6)	0.0171 (3)	
H8A	0.9591	−0.0345	0.0965	0.026*	
H8B	0.8903	−0.1193	0.0494	0.026*	
H8C	0.7391	−0.0709	0.0837	0.026*	
C9	1.0911 (2)	0.4117 (2)	0.13428 (5)	0.0138 (3)	
H9A	1.0178	0.5160	0.1349	0.017*	
H9B	1.1859	0.4248	0.1123	0.017*	
C10	1.1903 (2)	0.3783 (2)	0.18030 (5)	0.0134 (3)	
H10A	1.2682	0.2768	0.1793	0.016*	
H10B	1.2755	0.4719	0.1894	0.016*	
C11	0.8135 (2)	0.2553 (2)	0.15357 (5)	0.0123 (3)	
H11A	0.7264	0.1626	0.1448	0.015*	
H11B	0.7382	0.3586	0.1535	0.015*	
C12	0.9092 (2)	0.2244 (2)	0.20022 (5)	0.0136 (3)	
H12A	0.8123	0.2206	0.2220	0.016*	
H12B	0.9739	0.1156	0.2009	0.016*	
Cl1	0.09333 (5)	0.80876 (5)	0.181490 (13)	0.01515 (8)	
Cl2	0.52791 (5)	0.64529 (5)	0.132584 (12)	0.01449 (8)	
Cl3	0.34586 (5)	1.07346 (5)	0.105858 (12)	0.01456 (8)	
Cl4	0.55737 (5)	0.97161 (5)	0.219958 (12)	0.01489 (8)	
N1	0.95988 (18)	0.27010 (16)	0.12030 (4)	0.0100 (2)	
H1A	1.034 (3)	0.180 (2)	0.1220 (7)	0.012*	
N2	1.04987 (18)	0.35667 (16)	0.21386 (4)	0.0115 (2)	
H2A	0.9874	0.4552	0.2171	0.017*	
H2B	1.1124	0.3297	0.2412	0.017*	
O1	0.77955 (16)	0.59834 (15)	0.22753 (4)	0.0141 (2)	
H1C	0.721 (3)	0.632 (3)	0.2039 (7)	0.037 (7)*	
H1B	0.691 (3)	0.564 (3)	0.2421 (8)	0.033 (7)*	
Zn1	0.38078 (2)	0.86992 (2)	0.158284 (6)	0.01076 (5)	

### Atomic displacement parameters (Å^2^)

**Table:**

	*U*^11^	*U*^22^	*U*^33^	*U*^12^	*U*^13^	*U*^23^
C1	0.0112 (6)	0.0153 (7)	0.0071 (6)	−0.0005 (5)	0.0002 (5)	−0.0015 (5)
C2	0.0120 (6)	0.0152 (7)	0.0118 (7)	−0.0001 (5)	0.0007 (5)	−0.0004 (5)
C3	0.0101 (6)	0.0200 (8)	0.0125 (7)	0.0021 (5)	0.0017 (5)	0.0024 (6)
C4	0.0137 (7)	0.0264 (8)	0.0094 (7)	0.0002 (6)	0.0001 (5)	−0.0008 (6)
C5	0.0168 (7)	0.0205 (8)	0.0123 (7)	−0.0009 (6)	−0.0010 (5)	−0.0051 (6)
C6	0.0122 (7)	0.0153 (7)	0.0119 (7)	−0.0006 (5)	0.0006 (5)	−0.0023 (5)
C7	0.0152 (7)	0.0236 (8)	0.0156 (7)	0.0042 (6)	0.0011 (6)	0.0058 (6)
C8	0.0211 (8)	0.0133 (7)	0.0166 (7)	−0.0024 (6)	−0.0002 (6)	−0.0032 (6)
C9	0.0160 (7)	0.0142 (7)	0.0106 (6)	−0.0057 (5)	−0.0021 (5)	−0.0001 (5)
C10	0.0121 (6)	0.0174 (7)	0.0104 (6)	−0.0028 (5)	−0.0003 (5)	−0.0005 (5)
C11	0.0114 (6)	0.0163 (7)	0.0092 (6)	−0.0015 (5)	0.0013 (5)	−0.0012 (5)
C12	0.0167 (7)	0.0137 (7)	0.0101 (6)	−0.0043 (5)	0.0005 (5)	0.0002 (5)
Cl1	0.01373 (16)	0.01711 (17)	0.01506 (17)	−0.00208 (13)	0.00366 (12)	−0.00155 (13)
Cl2	0.01657 (17)	0.01327 (16)	0.01348 (16)	0.00368 (13)	0.00053 (12)	−0.00140 (12)
Cl3	0.01723 (17)	0.01358 (16)	0.01277 (16)	0.00171 (13)	0.00079 (12)	0.00279 (12)
Cl4	0.01381 (16)	0.01768 (17)	0.01260 (16)	−0.00089 (13)	−0.00196 (12)	−0.00211 (13)
N1	0.0111 (5)	0.0108 (6)	0.0078 (5)	0.0000 (4)	−0.0004 (4)	−0.0009 (4)
N2	0.0139 (6)	0.0122 (6)	0.0083 (5)	0.0003 (4)	0.0003 (4)	−0.0007 (4)
O1	0.0130 (5)	0.0165 (5)	0.0126 (5)	0.0005 (4)	0.0006 (4)	0.0012 (4)
Zn1	0.01151 (9)	0.01048 (9)	0.01017 (9)	0.00058 (6)	0.00039 (6)	−0.00003 (6)

### Geometric parameters (Å, °)

**Table:**

C1—C2	1.387 (2)	C9—H9B	0.9900
C1—C6	1.396 (2)	C10—N2	1.490 (2)
C1—N1	1.4892 (18)	C10—H10A	0.9900
C2—C3	1.396 (2)	C10—H10B	0.9900
C2—H2	0.9500	C11—N1	1.5096 (19)
C3—C4	1.395 (2)	C11—C12	1.516 (2)
C3—C7	1.504 (2)	C11—H11A	0.9900
C4—C5	1.387 (2)	C11—H11B	0.9900
C4—H4	0.9500	C12—N2	1.4923 (19)
C5—C6	1.395 (2)	C12—H12A	0.9900
C5—H5	0.9500	C12—H12B	0.9900
C6—C8	1.506 (2)	Cl1—Zn1	2.2702 (5)
C7—H7A	0.9800	Cl2—Zn1	2.2619 (4)
C7—H7B	0.9800	Cl3—Zn1	2.2690 (4)
C7—H7C	0.9800	Cl4—Zn1	2.2857 (4)
C8—H8A	0.9800	N1—H1A	0.901 (15)
C8—H8B	0.9800	N2—H2A	0.9200
C8—H8C	0.9800	N2—H2B	0.9200
C9—N1	1.5086 (19)	O1—H1C	0.832 (17)
C9—C10	1.512 (2)	O1—H1B	0.842 (16)
C9—H9A	0.9900		
			
C2—C1—C6	123.19 (13)	N2—C10—H10A	109.5
C2—C1—N1	119.34 (13)	C9—C10—H10A	109.5
C6—C1—N1	117.47 (13)	N2—C10—H10B	109.5
C1—C2—C3	119.92 (15)	C9—C10—H10B	109.5
C1—C2—H2	120.0	H10A—C10—H10B	108.1
C3—C2—H2	120.0	N1—C11—C12	110.09 (12)
C4—C3—C2	117.78 (15)	N1—C11—H11A	109.6
C4—C3—C7	121.85 (14)	C12—C11—H11A	109.6
C2—C3—C7	120.37 (15)	N1—C11—H11B	109.6
C5—C4—C3	121.21 (14)	C12—C11—H11B	109.6
C5—C4—H4	119.4	H11A—C11—H11B	108.2
C3—C4—H4	119.4	N2—C12—C11	111.49 (12)
C4—C5—C6	121.95 (15)	N2—C12—H12A	109.3
C4—C5—H5	119.0	C11—C12—H12A	109.3
C6—C5—H5	119.0	N2—C12—H12B	109.3
C5—C6—C1	115.80 (14)	C11—C12—H12B	109.3
C5—C6—C8	119.83 (14)	H12A—C12—H12B	108.0
C1—C6—C8	124.34 (14)	C1—N1—C9	114.53 (12)
C3—C7—H7A	109.5	C1—N1—C11	112.33 (11)
C3—C7—H7B	109.5	C9—N1—C11	108.80 (11)
H7A—C7—H7B	109.5	C1—N1—H1A	106.4 (13)
C3—C7—H7C	109.5	C9—N1—H1A	104.7 (13)
H7A—C7—H7C	109.5	C11—N1—H1A	109.6 (13)
H7B—C7—H7C	109.5	C10—N2—C12	111.83 (12)
C6—C8—H8A	109.5	C10—N2—H2A	109.3
C6—C8—H8B	109.5	C12—N2—H2A	109.3
H8A—C8—H8B	109.5	C10—N2—H2B	109.3
C6—C8—H8C	109.5	C12—N2—H2B	109.3
H8A—C8—H8C	109.5	H2A—N2—H2B	107.9
H8B—C8—H8C	109.5	H1C—O1—H1B	102 (2)
N1—C9—C10	109.95 (12)	Cl2—Zn1—Cl3	111.651 (16)
N1—C9—H9A	109.7	Cl2—Zn1—Cl1	112.737 (17)
C10—C9—H9A	109.7	Cl3—Zn1—Cl1	108.974 (16)
N1—C9—H9B	109.7	Cl2—Zn1—Cl4	109.040 (16)
C10—C9—H9B	109.7	Cl3—Zn1—Cl4	108.388 (17)
H9A—C9—H9B	108.2	Cl1—Zn1—Cl4	105.803 (16)
N2—C10—C9	110.52 (12)		
			
C6—C1—C2—C3	−3.1 (2)	N1—C9—C10—N2	−58.83 (17)
N1—C1—C2—C3	176.62 (13)	N1—C11—C12—N2	56.18 (17)
C1—C2—C3—C4	−0.4 (2)	C2—C1—N1—C9	−31.94 (19)
C1—C2—C3—C7	179.62 (14)	C6—C1—N1—C9	147.82 (14)
C2—C3—C4—C5	2.8 (2)	C2—C1—N1—C11	92.83 (16)
C7—C3—C4—C5	−177.28 (15)	C6—C1—N1—C11	−87.40 (16)
C3—C4—C5—C6	−1.7 (3)	C10—C9—N1—C1	−172.56 (12)
C4—C5—C6—C1	−1.7 (2)	C10—C9—N1—C11	60.82 (16)
C4—C5—C6—C8	176.45 (15)	C12—C11—N1—C1	172.87 (12)
C2—C1—C6—C5	4.1 (2)	C12—C11—N1—C9	−59.25 (16)
N1—C1—C6—C5	−175.63 (13)	C9—C10—N2—C12	55.24 (17)
C2—C1—C6—C8	−173.91 (15)	C11—C12—N2—C10	−54.17 (17)
N1—C1—C6—C8	6.3 (2)		

### Hydrogen-bond geometry (Å, °)

**Table:**

*D*—H···*A*	*D*—H	H···*A*	*D*···*A*	*D*—H···*A*
N1—H1A···Cl3^i^	0.90 (2)	2.46 (2)	3.2300 (14)	144 (2)
N2—H2A···O1	0.92	1.92	2.7925 (17)	157
N2—H2B···O1^ii^	0.92	2.19	2.9145 (17)	135
N2—H2B···Cl4^ii^	0.92	2.77	3.3965 (14)	127
N2—H2B···Cl1^iii^	0.92	2.85	3.4036 (14)	120
O1—H1C···Cl2	0.83 (2)	2.43 (2)	3.2361 (12)	164 (2)
O1—H1B···Cl4^iii^	0.84 (2)	2.31 (2)	3.1518 (13)	178 (3)

Symmetry codes: (i) *x*+1, *y*−1, *z*; (ii) −*x*+2, *y*−1/2, −*z*+1/2; (iii) −*x*+1, *y*−1/2, −*z*+1/2.
